# Induced Circularly Polarized Luminescence of Dynamically Racemic Luminophores Assisted by Formation of Helical Phase via Self-Assembly of Chiral Block Copolymers

**DOI:** 10.1021/acs.macromol.5c01122

**Published:** 2025-07-22

**Authors:** Chin-Cheng Kuo, Sheng-Wei Shao, Puhup Puneet, Eiji Yashima, Rong-Ming Ho

**Affiliations:** Department of Chemical Engineering, 34881 National Tsing Hua University No. 101, Section 2, Kuang-Fu Road, Hsinchu 30013, Taiwan

## Abstract

This work aims to suggest a facile approach for induced circular dichroism (ICD) and induced circularly polarized luminescence (iCPL) of the optically inactive, dynamically racemic luminophore tris­(1,1,1,2,2,3,3-heptafluoro-7,7-dimethyl-4,6-octanedionato)­europium­(III) (*rac*-Eu­(fod)_3_), through deracemization by interaction with chiral block copolymers (BCPs*), polystyrene-*b*-poly­(l-lactide) (PS-*b*-PLLA), and polystyrene-*b*-poly­(d-lactide) (PS-*b*-PDLA)). In a dilute solution, *rac*-Eu­(fod)_3_ exhibits no circular dichroism (CD) and a negligibly weak CPL in the presence of PS-*b*-PLLA and PS-*b*-PDLA. By contrast, significantly strong CD and CPL of the *rac*-Eu­(fod)_3_ chromophore can be induced by the mesochiral self-assembly of BCPs* with the formation of a helical phase (H*) that mostly relies on a highly enantiomer-selective deracemization of the *rac*-Eu­(fod)_3_ with propeller chirality (Δ or Λ form) upon complexation with the BCPs*, resulting in a high-luminescence dissymmetry factor up to approximately 10^–2^. The vibrational circular dichroism (VCD) results reveal that the VCD signals of the chiral polylactide chains in H* can be remarkably enhanced upon complexation with the deracemized Eu­(fod)_3_. The enhanced twisting of the packing of polylactide chains via interchain chiral interaction due to the presence of the nonracemic (deracemized) Eu­(fod)_3_ provides a unique platform for the design of chiroptical devices in various applications.

## Graphical abstract

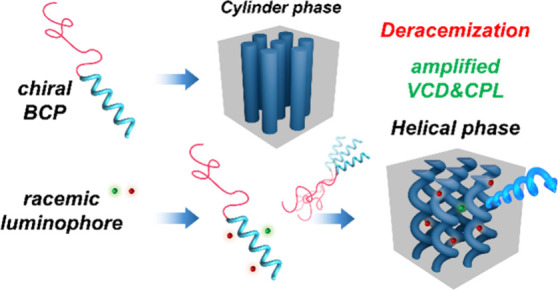

## Introduction

Chiral materials capable of emitting circularly polarized luminescence (CPL) have garnered intensive attention due to their promising applications in engineering devices such as 3D displays,^1,2^ data storage,^3^ chiroptical sensors,^4^ organic light emitting diodes,^5,6^ and molecular probe.^7,8^ Common strategies to achieve CPL activity typically involve the use of π-conjugated chiral small molecules,^9,10^ polymers,^11,12^ and coordination complexes,^13^ however, most of which exhibit low dissymmetry factors of emission (*g*_lum_), quantified as *g*_lum_ = 2­(*I*_L_–*I*_R_)/(*I*_L_ + *I*_R_), where *I*_L_ and *I*_R_ are the intensity of the left- and right-handed circularly polarized emissions, respectively. Although organic chemistry offers diverse methods for synthesizing enantiopure luminophores, the overall strategies are often daunting due to the intricate processes involved in both synthesis and chiral separation. Yashima and co-workers demonstrated the feasibility of inducing one-handed helicity in dynamically racemic helical polyacetylenes through noncovalent interactions with chiral dopants. This interaction results in an induced electric circular dichroism (ICD) in the π-conjugated polymer backbones and represents an efficient strategy for inducing one-handed helicity via noncovalent host–guest interactions.^14,15^ ` Following similar strategies, several pioneering studies investigated the emergence of induced circularly polarized luminescence (iCPL) in achiral or dynamically racemic lanthanide complexes through coordination with chiral ligands^16–19^ or in chiral solvents.^19,20^ Interestingly, the CPL activity arising from the chiral lanthanide complexes is particularly compelling due to their attractive emission characteristics that include large Stokes shifts, long emission lifetimes, narrow emission bands with low full width at half-maximum (fwhm), and high quantum yields.^21^ However, the manipulation of lanthanide ion coordination spheres remains a formidable challenge due to their high kinetic lability and lack of pronounced stereochemical preferences. Recently, the co-assembly of chiral and achiral luminophores has been extensively employed to enhance or induce CPL activity through the construction of hierarchical textures or embedded within chiral nematic liquid crystals^22^ and an achiral π-conjugated polymer matrix.^23^ Notably, these studies explicitly indicate that well-ordered self-assembled nanostructure formations, such as a one-handed helical assembly, can lead to significant amplification of chiroptical properties in the photoexcited state for CPL activities.

Block copolymers (BCPs) are renowned for their capacity to self-assemble into various ordered phases, including spheres (S), hexagonally packed cylinders (HC), double gyroids (DG), and lamellae (L) with nanostructured textures.^24–26^ The self-assembly of BCPs is driven by the microphase separation of the constituent blocks that is influenced by secondary interactions. The resulting phase morphology is primarily determined by the product of the interaction parameter (χ) and the degree of polymerization (*N*), termed χ*N*, along with the compositional asymmetry of the blocks. A helical phase (H*) with controlled helical sense has been discovered during the self-assembly of enantiomeric BCPs (referred to as chiral block copolymers (BCPs*)),^27,28^ polystyrene-*b*-poly­(l-lactide) (PS-*b*-PLLA) or polystyrene-*b*-poly­(d-lactide) (PS-*b*-PDLA) (Figure 1a) when the volume fraction of PLLA (*f*_PLLA_^v^) was in the range of 0.3–0.4, resulting from chirality transfer from molecular chirality to mesoscale phase chirality in the self-assembled polymer systems. The chiral blocks (PLLA or PDLA) favorably stack from each other in a left- or right-handed direction in the PS matrix, respectively, via twisting and shifting mechanism, thus producing one-handed nanohelices.^29,30^ Such a unique helical phase could also be induced in a helix-sense-selective manner using achiral block copolymers when chiral dopants were embedded within the self-assembled achiral block copolymers.^31^ This strategy has been applied to enhance a CPL activity of chiral luminescent dopants.^32,33^

**Figure 1:**
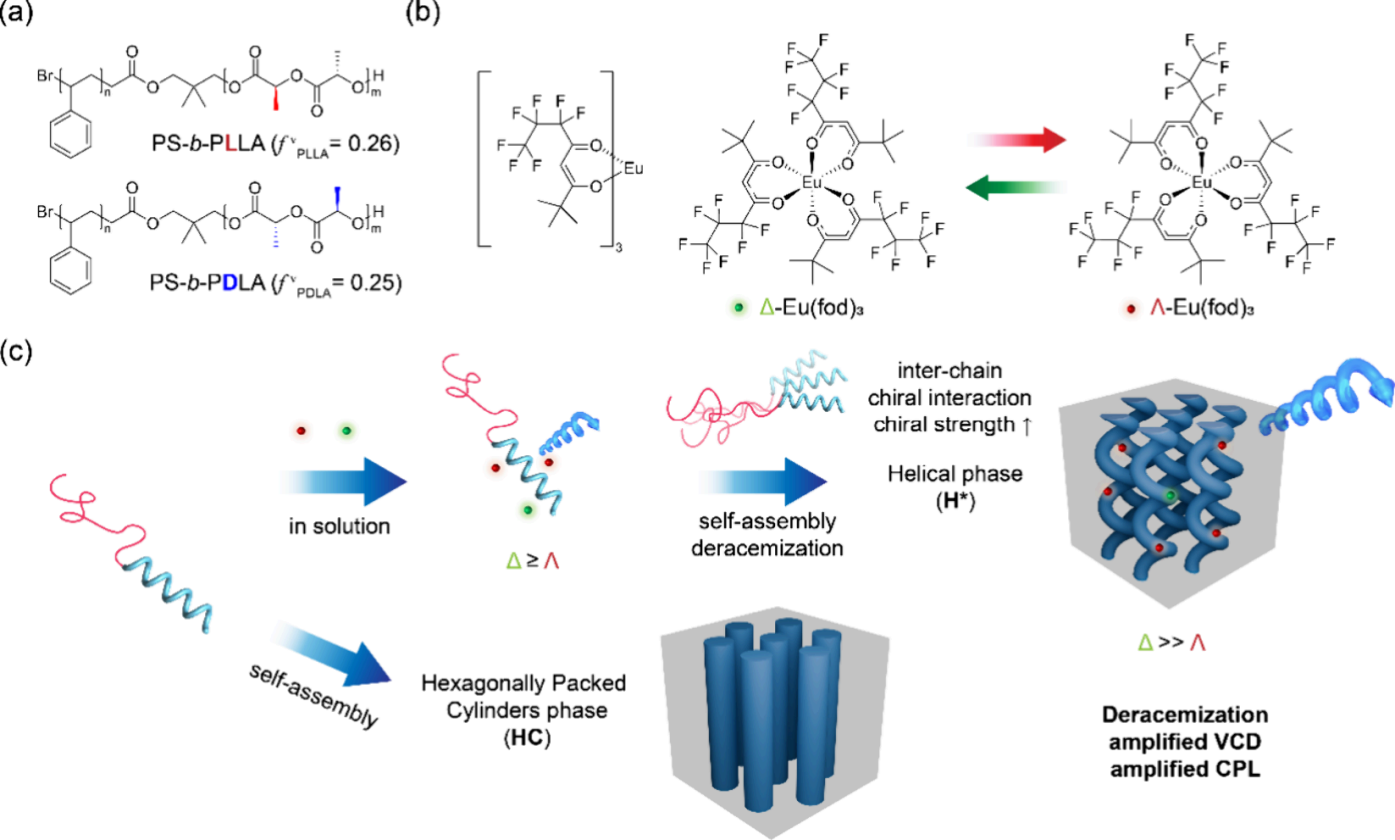
Chemical structures of chiral block copolymers (BCP*), PS-*b*-PLLA, and PS-*b*-PDLA (a), and a dynamically racemic luminophore, *rac*-Eu­(fod)_3_, composed of an equal mixture of kinetically labile Δ and Λ enantiomers under equilibrium (b). (c) Schematic illustrations of the hexagonally packed cylinder phase (HC) for the self-assembly of PS-*b*-PLLA (*f*_PLLA_^*v*^ = 0.26) in the absence of *rac*-Eu­(fod)_3_ and hierarchical asymmetric transformation of a racemic mixture of Eu­(fod)_3_ into a nonracemic one upon complexation with the chiral PLLA segment in solution and further embedded within the self-assembled helical phase (H*), resulting in the emergence of amplified ICD and iCPL mediated by a highly enantiomer-selective deracemization of the *rac*-Eu­(fod)_3_ in the H* phase.

This work aims to explore helically stacked chiral polymer segments, such as PLLA and PDLA chains in BCPs*, with the formation of H* phase as a versatile host, optimally suited for the incorporation of achiral and chiral luminophores even yet kinetically labile racemic ones, through secondary interactions with the luminophores as guests for ICD and iCPL. To this end, a dynamically racemic lanthanide complex, tris­(1,1,1,2,2,3,3-heptafluoro-7,7-dimethyl-4,6-octane dionato)­europium­(III) (*rac*-Eu­(fod)_3_), was utilized as a typical luminophore composed of an equal mixture of kinetically labile Δ and Λ enantiomers (Figure 1b) for the examination of the impact of helically assembled chiral PLLA and PDLA segments in the H* with exclusive handedness on the emergence and further amplification of chiroptical activities of *rac*-Eu­(fod)_3_ due to the asymmetric transformation into a nonracemic one (deracemization) assisted by the formation of the helical phase via enhanced chiral strength (i.e., interchain chiral interaction) (Figure 1c).

## Results and Discussion

To achieve the chirality transfer from BCP* to a racemic luminophore, *rac*-Eu­(fod)_3_ for the induction of optical activities, it is essential to create the association between chiral polylactide in BCP* and *rac*-Eu­(fod)_3_. Owing to its highly coordinated complexation characteristics of europium­(III) (Eu^3+^) ion, the polar carbonyl groups (C=O) with lone pairs of the chiral polylactide segment in BCP*, is anticipated to associate with an europium­(III) (Eu^3+^) ion, giving the formation of chelated complex via coordination bonding. As shown in the ^1^H-NMR spectrum (Figure 2a), the characteristic singlet, corresponding to the CH_3_ groups of *rac*-Eu­(fod)_3_, shows an upfield shifting from 2.71 to 1.88 ppm in the presence of PS-*b*-PLLA. On the other hand, the CH groups of the PLLA segment are shifted to downfield with *rac*-Eu­(fod)_3_ (10 mol %) (Figure 2b). To further evidence the proposed association, ^13^C NMR experiments were conducted. As shown in Supporting Information Figure S1, after doping with Eu­(fod)_3_, the signal of the carbonyl carbon shifts downfield from 169.58 to 169.63 ppm, indicating the coordination of carbonyl groups with Eu­(fod)_3_. Those observations are consistent with the expected association between PS-*b*-PLLA and *rac*-Eu­(fod)_3_. Similar results can also be found in PS-*b*-PDLA/(Eu­(fod)_3_)_0.1_ (subscript denoted as the molar ratio of [Eu­(fod)_3_]:[LA]) (Figure 2a,b). There is almost no chemical shift difference in the aromatic regions of the PS segment in BCP* whether the sequence is with or without *rac*-Eu­(fod)_3_ (Figure 2c).

**Figure 2:**
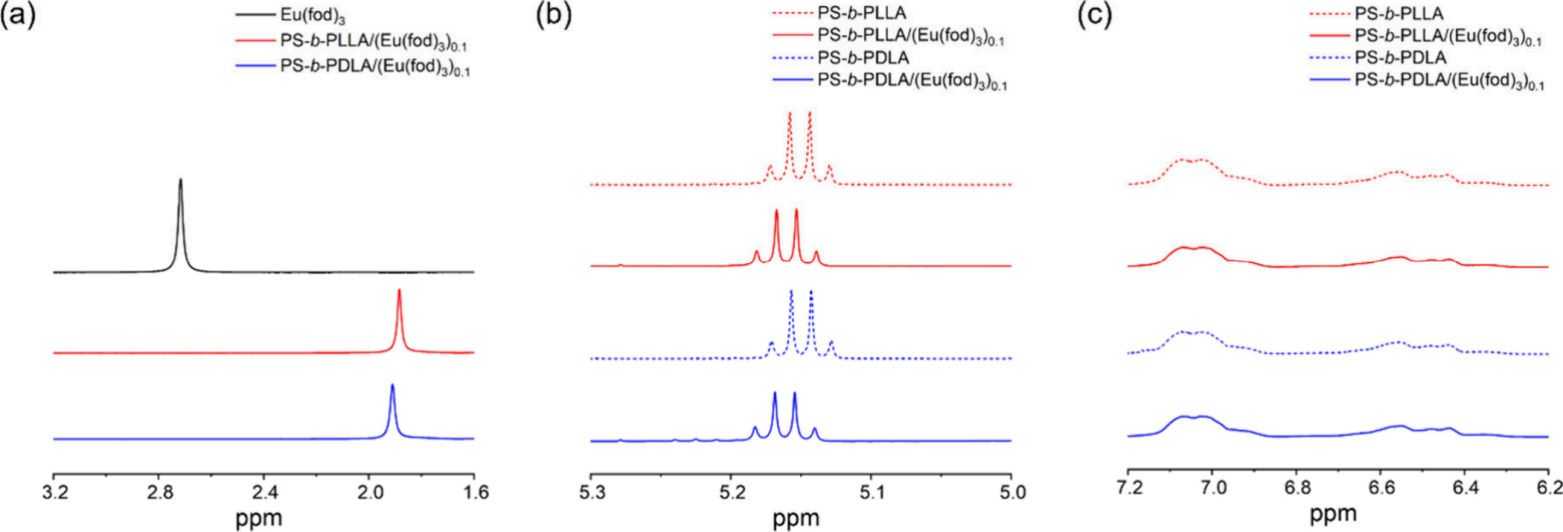
^1^H NMR spectra of (a) Eu­(fod)_3_, PS-*b*-PLLA/(Eu­(fod)_3_)_0.1_, and PS-*b*-PDLA/(Eu­(fod)_3_)_0.1_ in CDCl_3_ (3.2–1.6 ppm); (b) those of PS-*b*-PLLA, PS-*b*-PLLA/(Eu­(fod)_3_)_0.1_, PS-*b*-PDLA, and PS-*b*-PDLA/(Eu­(fod)_3_)_0.1_ in CDCl_3_ (5.3–5.0 ppm); (c) those of PS-*b*-PLLA, PS-*b*-PLLA/(Eu­(fod)_3_)_0.1_, PS-*b*-PDLA, and PS-*b*-PDLA/(Eu­(fod)_3_)_0.1_ in CDCl_3_ (7.2–6.2 ppm).

PS-*b*-PLLA or PS-*b*-PDLA BCPs* with the volume fraction of PLLA (*f*_PLLA_^v^) ranging from 0.3 to 0.4 self-assembles as the H* phase, whereas the HC phase is formed for the volume fraction ranging from 0.2 to 0.3.^34,35^ Figure 3a shows the transmission electron microscopy (TEM) projection image of the HC-forming PS-*b*-PLLA (*f*_PLLA_^v^ = 0.26) where the dark region represents the PS matrix selectively stained by RuO_4_ and the bright region corresponds to the PLLA microdomain. One-dimensional (1D) small-angle X-ray scattering (SAXS) profile with reflections at the relative *q* values of 1:√4:√9 (Figure 3c) suggests the formation of hexagonal packing. Based on the TEM and SAXS results, self-assembled PS-*b*-PLLA is referred to as a phase with hexagonally packed cylinders as anticipated from the *f*_PLLA_^v^ value (Figure 1c). Interestingly, in the presence of 10 mol % *rac*-Eu­(fod)_3_, the complex (PS-*b*-PLLA/(Eu­(fod)_3_)_0.1_) was found to form a dark helical microdomain as supported by its TEM image without any staining (Figure 3b); namely, there is an inverted contrast with dark PLLA helices in bright PS matrix as compared to Figure 3a. This observation further evidences the presence of europium metals preferentially associated with the one-handed helical polylactide blocks, giving mass contrast through complexation. The corresponding 1D SAXS profile with the reflections at the relative *q* values of 1:√4:√7 indicates the formation of hexagonally packed helices (H*). The primary scattering peak at the low-*q* region shifts from 0.207 to 0.181 nm^–1^, suggesting that the domain spacing is enlarged by the preferential association with *rac*-Eu­(fod)_3_ due to the enlargement of segregation strength. As a result, the phase transition from the achiral HC to chiral H* phase can be achieved by introducing racemic Eu­(fod)_3_ due to the change in the constituted volume fractions of the PS-*b*-PLLA by coordinating the *rac*-Eu­(fod)_3_ to the PLLA block (Figure 1c). A similar H* phase formation can also be observed in self-assembled PS-*b*-PDLA in the presence of *rac*-Eu­(fod)_3_ (PS-*b*-PDLA/(Eu­(fod)_3_)_0.1_) (Figure 3d–f).

**Figure 3:**
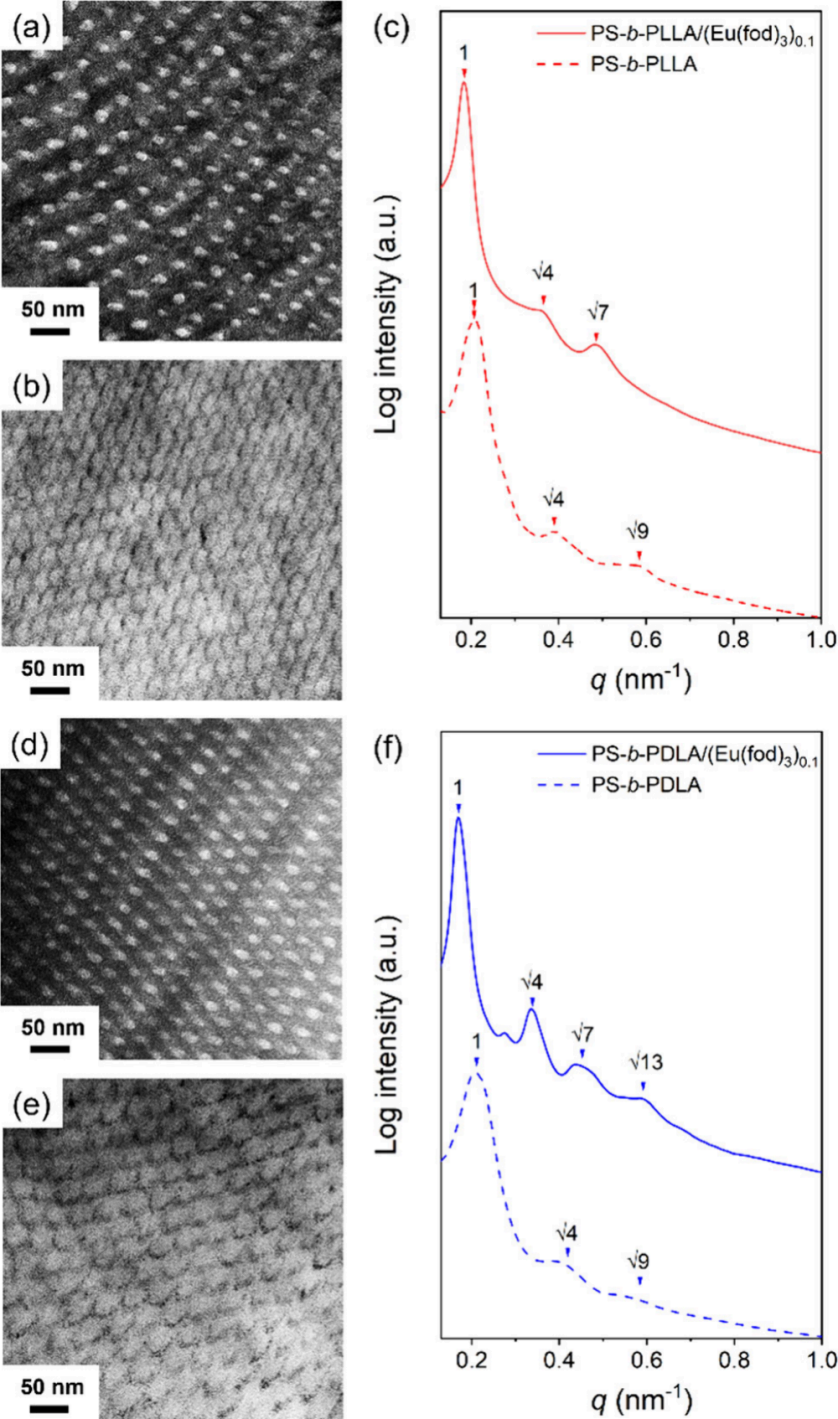
(a) TEM micrograph of the HC phase from self-assembled PS-*b*-PLLA with RuO_4_ staining; (b) TEM micrograph of the H* phase from self-assembled PS-*b*-PLLA/(Eu­(fod)_3_)_0.1_ without staining; (c) corresponding 1D SAXS profiles; (d) TEM micrograph of the HC phase from self-assembled PS-*b*-PDLA with RuO_4_ staining; (e) TEM micrograph of the H* phase from self-assembled PS-*b*-PDLA/(Eu­(fod)_3_)_0.1_ without staining; (f) corresponding 1D SAXS profiles.

To assess the feasibility for the iCPL of dynamically racemic *rac*-Eu­(fod)_3_ induced by BCP*, CD and absorption spectra of *rac*-Eu­(fod)_3_ in the absence and presence of PS-*b*-PLLA and PS-*b*-PDLA were first measured to investigate the effect of the chiral polylactide-based BCP* on the ICD of the *rac*-Eu­(fod)_3_. As shown in Figure 4a, in the dilute dichloromethane (DCM) solutions of PS-*b*-PLLA/(Eu­(fod)_3_)_0.1_ and PS-*b*-PDLA/(Eu­(fod)_3_)_0.1_ as well as *rac*-Eu­(fod)_3_, no recognized CD signal from the absorption with respect to the π–π* transition of three fod ligand moieties attached to the Eu^3+^ ion can be found. By contrast, relatively strong bisignate CD signals can be found in the thin-film state of PS-*b*-PLLA/(Eu­(fod)_3_)_0.1_ and PS-*b*-PDLA/(Eu­(fod)_3_)_0.1_ before the formation of the well-ordered H* phase (i.e., disordered state) (dashed lines in Figure 4b). The emergence of these bisignate exciton-coupled CD bands is attributed to the asymmetric transformation of *rac*-Eu­(fod)_3_ into a nonracemic one, namely, by deracemizations of *rac*-Eu­(fod)_3_ in an enantioselective way through interaction with chiral polylactide chain. The positive and negative exciton couplet CDs observed in the PS-*b*-PLLA/(Eu­(fod)_3_)_0.1_ and PS-*b*-PDLA/(Eu­(fod)_3_)_0.1_ can be assigned to Λ- and Δ-configurations enriched in the deracemized Eu­(fod)_3_, respectively, based on the literature.^36^ Accordingly, the association of chiral polylactide block with *rac*-Eu­(fod)_3_ in the solution state might be too weak to give ICD signals for the *rac*-Eu­(fod)_3_ but the ICD signals can be enhanced in the solid state by the aggregation of the BCP* chain associated with (Eu­(fod)_3_)_0.1_. With the formation of the H* phase, it is instinctive to expect the significant enhancement of the interchain chiral interaction, giving the profound amplification of the ICD signals (solid lines in Figure 4b). The helical phase formation of one-handed polylactide-based BCPs* appears to be indispensable for the emergence of such strong CD signals, resulting from deracemization of *rac*-Eu­(fod)_3_ assisted by the interchain chiral interactions of polylactide chains.

**Figure 4:**
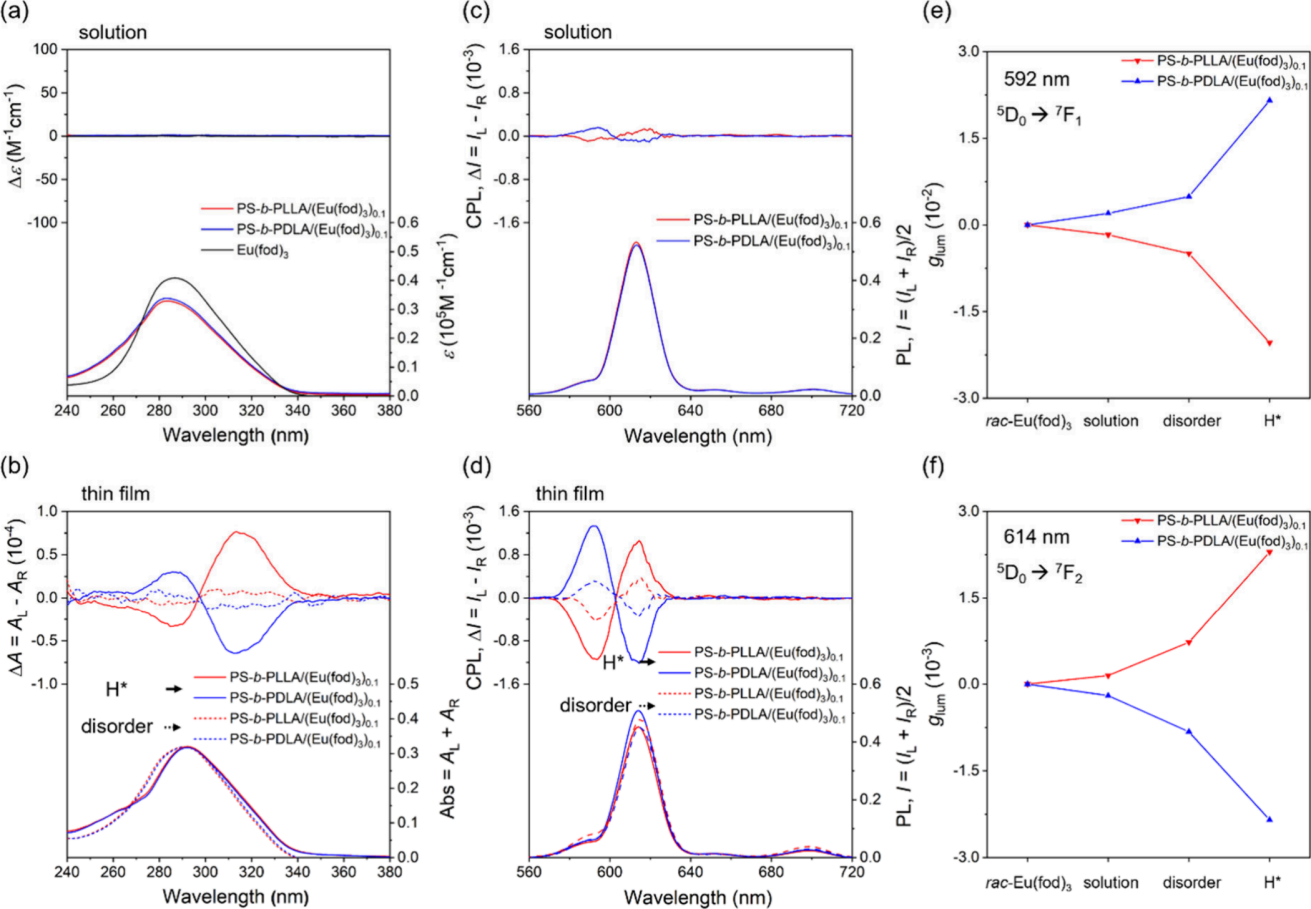
(a) CD and absorption spectra of PS-*b*-PLLA/(Eu­(fod)_3_)_0.1_, PS-*b*-PDLA/(Eu­(fod)_3_)_0.1_, and *rac*-Eu­(fod)_3_ in DCM ([Eu­(fod)_3_)] = 1.2 mM); (b) CD and absorption spectra of PS-*b*-PLLA/(Eu­(fod)_3_)_0.1_ and PS-*b*-PDLA/(Eu­(fod)_3_)_0.1_ in the thin-film state with (solid lines) and without (dashed lines) the formation of the H* phase. CPL and PL spectra of PS-*b*-PLLA/(Eu­(fod)_3_)_0.1_ and PS-*b*-PDLA/(Eu­(fod)_3_)_0.1_ in DCM ([Eu­(fod)_3_)] = 4.8 × 10^–2^ M) (c) and those in the solid state with the disordered phase (dashed lines) and with the formation of H* phase (solid lines) (d). Comparison of the *g*_lum_ value for the ^5^D_0_ → ^7^F_1_ transition (e) and ^5^D_0_ → ^7^F_2_ transition (f).

The deracemization of *rac*-Eu­(fod)_3_ with the chiral interaction from self-assembly is expected to induce CPL at the photoexcited state based on the ICD results of Figure 4a. Figure 4c shows the CPL and photoluminescence (PL) spectra of PS-*b*-PLLA/(Eu­(fod)_3_)_0.1_ and PS-*b*-PDLA/(Eu­(fod)_3_)_0.1_in DCM. Among four characteristic emission bands of Eu­(fod)_3_ that appeared at 592, 613, 652, and 699 nm in the PL spectra, corresponding to the ^5^D_0_ → ^7^F_J_ (*J* = 1, 2, 3, 4) transitions, respectively, only the ^5^D_0_ → ^7^F_2_ and ^5^D_0_ → ^7^F_1_ transitions can be reasonably recognized. Note that the ^5^D_0_ → ^7^F_1_ transition is attributed to the character of the magnetic dipole moment transition, whereas the ^5^D_0_ → ^7^F_2_ transition is the character of the electronic dipole moment transition.^37^ Consequently, the luminescence dissymmetry factor (*g*_lum_) for the ^5^D_0_ → ^7^F_1_ transition will obviously be higher than that for the ^5^D_0_ → ^7^F_2_ transition.

As shown in Figure 4c, the PS-*b*-PLLA/(Eu­(fod)_3_)_0.1_ and PS-*b*-PDLA/(Eu­(fod)_3_)_0.1_ complexes both show weak mirror-image bisignate CPL bands that appear at 592 nm (^5^D_0_ → ^7^F_1_) and 613 nm (^5^D_0_ → ^7^F_2_) with a *g*_lum_ value in the order of 10^–3^ and 10^–4^, respectively, resulting from slightly deracemized Eu­(fod)_3_ mediated by intermolecular interactions with molecularly dispersed one-handed helical polylactide-based BCP chains in DCM. The correlation between the absolute configurations of optically active Eu­(III) complexes composed of chiral chelating ligands and the signs of the CPL signal has been well-documented. Therefore, the sample of PS-*b*-PLLA/(Eu­(fod)_3_)_0.1_ shows a negative CPL band for the ^5^D_0_ → ^7^F_1_ transition and a positive CPL band for the ^5^D_0_ → ^7^F_2_ transition; the slightly deracemized Eu­(fod)_3_ can be assigned to the Λ-configuration. On the other hand, PS-*b*-PDLA/(Eu­(fod)_3_)_0.1_ shows opposite CPL bands with an opposite Δ-configuration enriched in the deracemized Eu­(fod)_3_ (Figure 4c). These assignments are in good agreement with those estimated by the exciton-coupled CD results (Figure 4b).

In the thin-film state of the PS-*b*-PLLA/(Eu­(fod)_3_)_0.1_ and PS-*b*-PDLA/(Eu­(fod)_3_)_0.1_ before the formation of the well-ordered H* phase (i.e., disordered state), the CPL intensities increase slightly (dashed lines in Figure 4d) due to the asymmetric transformation of *rac*-Eu­(fod)_3_ into a nonracemic one through chiral interaction with one-handed helical polylactide chains as described above. With the formation of the H* phase, however, significantly strong CPL bands with the *g*_lum_ value up to approximately 10^–2^ (^5^D_0_ → ^7^F_1_ transition (592 nm)) and 10^–3^ (^5^D_0_ → ^7^F_2_ transition (613 nm)) can be observed in the self-assembled PS-*b*-PLLA/(Eu­(fod)_3_)_0.1_ and PS-*b*-PDLA/(Eu­(fod)_3_)_0.1_ (solid lines in Figure 4d), due to a highly enantiomer-selective deracemization of the *rac*-Eu­(fod)_3_ embedded in the H* phase as evidenced in the strong ICD in the H* phase (Figure 4b), giving approximately 1 order of magnitude more intensity than those in solution and in the disordered state as summarized in Figure 4e,f.

To investigate the influence of *rac*-Eu­(fod)_3_ on the helical conformation of PLLA and PDLA blocks of BCPs* in solution and in the self-assembled achiral HC and chiral H* phases, vibrational CD (VCD) and IR measurements were carried out. PS-*b*-PLLA and PS-*b*-PDLA exhibit mirror-imaged split-type Cotton effects in the C=O stretching vibration region at 1760 cm^–1^ (Figure 5a) and a series of mirror-imaged bands including those due to the C–O–C vibrations (Figure 5c) in CDCl_3_, suggesting that the PLLA and PDLA blocks possess left- and right-handed helical structures, respectively.^38^ In the presence of *rac*-Eu­(fod)_3_, the VCD signals almost remain unchanged in CDCl_3_ (Figure 5a,c), indicating that the association between BCPs* and *rac*-Eu­(fod)_3_ hardly affected the helical conformation of the PLLA and PDLA blocks of BCP* in solution.

**Figure 5:**
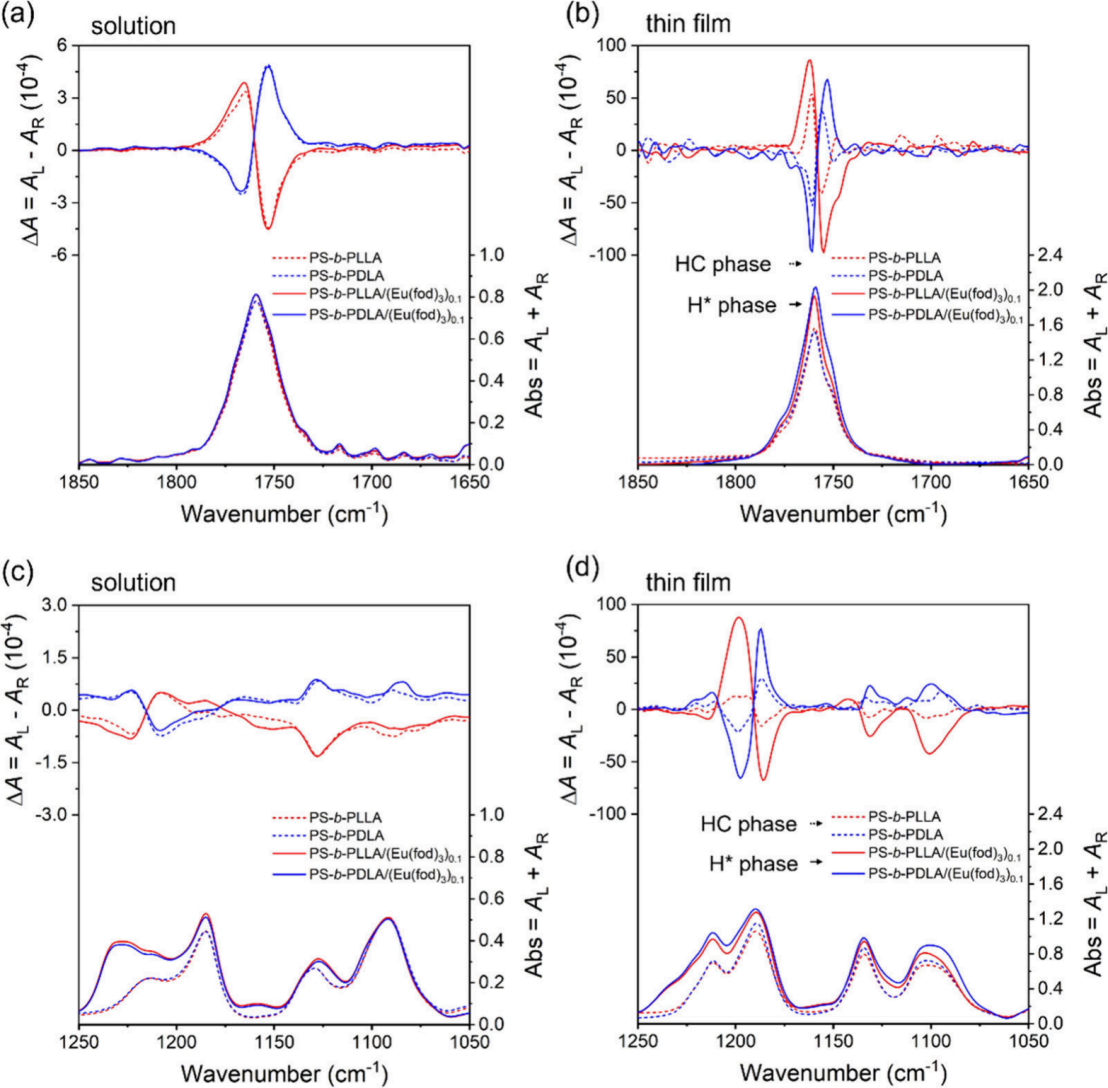
VCD and IR spectra of PS-*b*-PLLA, PS-*b*-PDLA, PS-*b*-PLLA/(Eu­(fod)_3_)_0.1_, and PS-*b*-PDLA/(Eu­(fod)_3_)_0.1_ in CDCl_3_ ([Eu­(fod)_3_)] = 9.6 × 10^–2^ M) (a and c) and in the solid state with the formation of achiral HC and chiral H* phases (b and d) in the C=O stretching (a and b) and C–O–C vibration regions (c and d).

As described above, PS-*b*-PLLA and PS-*b*-PDLA BCP* self-assemble to form an achiral HC phase (Figure 3a,d), which showed nearly mirror-imaged split-type VCDs approximately centered at 1760 cm^–1^ in the C=O stretching region as seen in solution, while the VCD intensities can be significantly enhanced in the HC phase (dashed lines in Figure 5b). Moreover, in sharp contrast to the VCDs in solution (Figure 5c), split-type bisignate VCDs clearly appear approximately at 1190 cm^–1^ in the C–O–C vibrational region of the PLLA and PDLA blocks of BCP* in the solid HC phase that are remarkably different from those in solution in both the intensity and pattern (dashed lines in Figure 5d). The observed variations are mostly attributed to the interchain chiral interaction between the PLLA and PDLA segments of BCP*, resulting from the microphase-separated domain formation composed of the PLLA and PDLA chains at mesoscale.

Surprisingly, in the presence of *rac*-Eu­(fod)_3_, further significantly enhanced split-type VCD signals appear in both the C=O stretching and C–O–C vibrational regions, resulting from the H* phase formation of the BCPs* (solid lines in Figure 5b,d). The observed notable enhancements in the VCD signals in both the C=O stretching and C–O–C vibrations in the chiral H* phase over in the achiral HC phase are most likely due to the helical arrangements of the polylactide chains in a one-handed helical array, resulting in the H* phase formation in the presence of *rac*-Eu­(fod)_3_, which further favorably deracemizes in a highly enantiomer-selective manner, thus providing a strong CPL induced by interactions with helically stacked homochiral polylactide chains in the H* phase.

## Conclusion

In conclusion, this work develops a simple and effective strategy to induce optical activities for dynamically racemic luminophores in the ground and photoexcited states through deracemization assisted by the helical phase (H*) from the self-assembly of one-handed helical polylactide-based BCPs*. Yet, note that the formation of H* may require specific conditions such as the appropriate compositions of constituted blocks in the block copolymer to achieve the induced CD for the racemic chromophore. By taking advantage of mesochiral self-assembly for the formation of well-ordered helical phase, strongly enhanced CD and iCPL activities could be achieved in the thin-film state due to interchain chiral interaction between the one-handed helical polylactide chains. The iCPL activity with large *g*_lum_ reaching to the order of 10^–2^ mostly attributed to a highly enantiomer-selective deracemization of *rac*-Eu­(fod)_3_ with preferential propeller chirality (Δ or Λ form) upon complexation with the homochiral polylactide chains packed in the H* phase, whereas solution state and disordered solid state exhibit negligible or weak CD and CPL signals. A key feature of this work is the proposed hierarchical amplification mechanism at which the one-handed polylactide-based BCPs* induce a very small enantiomeric imbalance in the kinetically labile enantiomers (Δ or Λ form) of racemic Eu­(fod)_3_ in solution through deracemization. Interestingly, the polylactide-based BCPs* complexed with such weakly deracemized Eu­(fod)_3_ (*derac-*Eu­(fod)_3_) are found to give the formation of the chiral H* phase from self-assembly instead of the achiral HC phase, and at the same time further enantioselective deracemization takes place, thus providing a significantly enhanced strong iCPL due to the *derac*-Eu­(fod)_3_ embedded in the H* phase, in which the polylactide-based BCPs* helically stack in a more twisting manner, giving amplified VCD signals due to intermolecularly associated chiral polylactide chains with single handedness. While this study highlights design principles for enhancing chiroptical signals, it should be noted that the observed effects rely on specific molecular interactions and the formation of a particular mesoscopic phase. The findings presented here have significant implications for various applications, suggesting that this approach could be used to create nanostructured monoliths with notable CPL activity. This might lead to new concepts in the design of optical devices with potential applications in optoelectronics, chiral sensing, enantioseparation, and asymmetric catalysis.

## Supplementary Material



## Notes

Additional information on sample preparation, instrumentation, and chiroptical measurements (PDF)
