# Supplementary material for: Induced Circularly Polarized Luminescence of Dynamically Racemic Luminophores Assisted by Formation of Helical Phase via Self-Assembly of Chiral Block Copolymers
Source: Macromolecules. 2025 Jul 22;58(15):8308–15. doi: 10.1021/acs.macromol.5c01122 (PMC12356058; doi:10.1021/acs.macromol.5c01122)
Supplement: Supplementary file 1 [file ma5c01122_si_001.pdf]

Supporting Information

Induced Circularly Polarized Luminescence of Dynamically Racemic Luminophores Assisted by Formation of Helical Phase *via* Self-assembly of Chiral Block Copolymers

Chin-Cheng Kuo [a‡], *Sheng-Wei Shao* [a‡], *Puhup Puneet* [a], *Eiji Yashima* [a], *Rong-Ming Ho* [a,*]

[a] Department of Chemical Engineering, National Tsing Hua University No. 101, Section 2, Kuang-Fu Road, Hsinchu, Taiwan 30013, R.O.C.

Keywords: chiral block copolymers, chirality transfer, induced circular dichroism, induced circularly polarized luminescence, mesochiral self-assembly, amplification of *g*-factor

‡ Equal contribution

*Corresponding authors

Email: rmho@mx.nthu.edu.tw

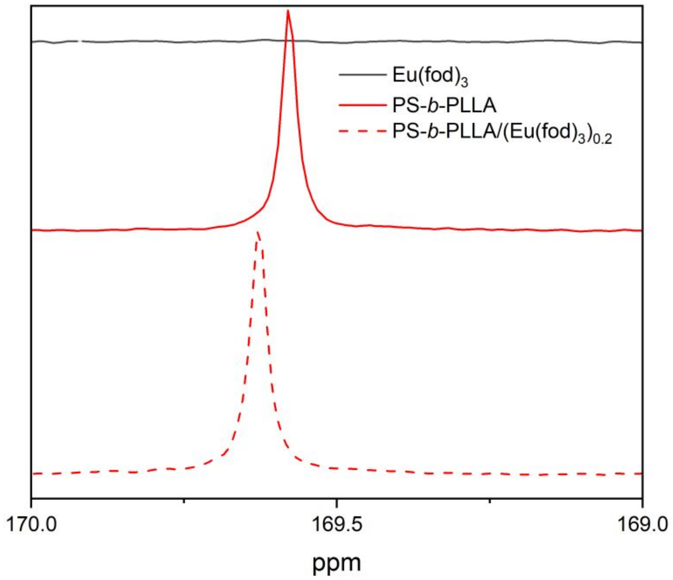

Figure S1. ^{13}C-NMR spectra of $Eu(fod)_3$, PS-*b*-PLLA and PS-*b*-PLLA/$(Eu(fod)_3)_{0.1}$ in $CDCl_3$.

EXPERIMENTAL

Synthesis of hydroxyl-capped polystyrene (PS-OH). The polystyrene-*block*-poly(L-lactide) (PS-*b*-PLLA) and polystyrene-*block*-poly(D-lactide) (PS-*b*-PDLA) chiral block copolymers (BCP*) were synthesized *via* two-step polymerization. First, as shown in **Figure S2a**, PS-OH was synthesized using double headed initiator (DHI), styrene as monomer and CuBr/HMTETA as catalyst through atom transfer radical polymerization (ATRP). The reaction mixture was stirred at 110 °C for 3 hrs and then quenched by ice. Subsequently, the crude sample was dissolved in dichloromethane (DCM) and followed by the precipitation in methanol (MeOH). The precipitate was filtered and dried under vacuum conditions to obtain the desired PS-OH. Further characterizations were examined by gel permeation chromatography (GPC) and nuclear magnetic resonance spectroscopy (^{1}H-NMR).

Synthesis of PS-*b*-PLLA and PS-*b*-PDLA. As shown in **Figure S2b**, PS-OH was further employed as a macroinitiator for controlled living ring-opening polymerization (ROP) of L-lactide in the presence of Tin(II) 2-ethylhexanoate ($Sn(Oct)_2$) as catalyst and toluene as solvent. The reaction mixture was stirred at 110 °C for 3 hrs and then quenched by ice. Subsequently, the crude sample was dissolved in dichloromethane (DCM) and followed by the precipitation in methanol (MeOH). The precipitate was filtered and dried under vacuum conditions to obtain the desired PS-*b*-PLLA. Further purification was carried out by dissolving the precipitate in acetonitrile (ACN) for fractionation to remove undesired PLLA homopolymer before characterization. The synthesis of PS-*b*-PDLA were carried out under the similar synthetic routes. Further characterizations were examined by gel permeation chromatography (GPC) and nuclear magnetic resonance spectroscopy (^{1}H-NMR). **Table S1** lists the PS-OH, PS-*b*-PLLA and PS-*b*-PDLA samples utilized in this experiment.

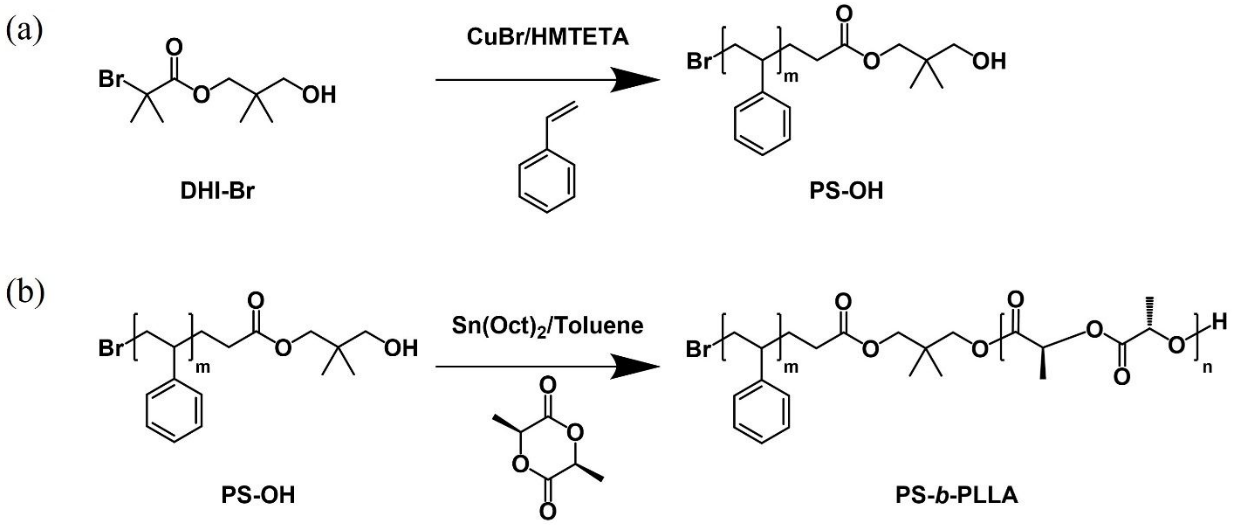

Figure S2. Synthetic routes of PS-*b*-PLLA by using two-step polymerization. Similar strategy was employed for the synthesis of PS-*b*-PDLA sample.

Table 1. Characterization of synthesized PS-OH, PS-*b*-PLLA and PS-*b*-PDLA samples.

Sample	Mn (g/mol)	Mn, $_{PS}$ (g/mol)	Mn, $_{PLLA/PDLA}$ (g/mol)	$f_{PS}^{v\,a}$	$f_{PLLA/PDLA}^{v\,a}$	$Đ^{b}$
PS-OH	26,000	26,000	-	-	-	1.08
PS-*b*-PLLA	36,900	26,000	10,900	0.74	0.26	1.14
PS-*b*-PDLA	36,500	26,000	10,500	0.75	0.25	1.13

[a] calculated from ^{1}H NMR based on, ρ_{ps}= 1.05 g/cm^3, ρ_{PLLA} =1.25 g/cm^3.

[b] determined by GPC.

Characterization of PS-OH, PS-*b*-PLLA and PS-*b*-PDLA. GPC with polystyrene standard calibration curve can be used to calculate the molecular weight and polydispersity index (Đ) of the synthetic sample. On the other hand, ^{1}H NMR can be used to determine the chemical structure and calculate the volume fraction of the synthetic sample. **Figure S3** show that the polydispersity of PS-OH is approximately 1.08, and the molecular weights of PS-OH are 26,000 g/mol. Those two results indicate that PS-OH is a suitable macroinitiator and ready for the synthesis of aimed chiral diblock copolymers with narrow molecular weight distribution. **Figure S4** show the ^{1}H NMR spectrum of PS-OH. ^{1}H NMR (500 MHz, $CDCl_3$): δ = 6.26-7.22 (PS, m, Ar-H, 5H), 1.82 (PS, s, backbone CH, 1H), 1.41 (PS, s, backbone CH_2, 2H) can be clearly identified, in line with the chemical structure of PS-OH as shown in **Figure S2**.

Figure S5 and **S6** show that the polydispersity of PS-*b*-PLLA and PS-*b*-PDLA is 1.14 and 1.13, respectively. The shifting of retention time can be found in the comparison of GPC curves between PS-OH and PS-*b*-PLLA or PS-*b*-PDLA, confirming the successful synthesis of the aimed chiral block copolymers. **Figure S6** and **S7** show the ^{1}H NMR spectrum of PS-*b*-PLLA and PS-*b*-PDLA. ^{1}H NMR spectrum of PS-PLLA and PS-PDLA assignments are as follows: ^{1}H NMR (500 MHz, $CDCl_3$): δ = 7.22-6.26 (PS, m, Ar-H, 5H), 5.16-5.12 (PLLA, q, CH, 1H), 1.82 (PS, s, backbone CH, 1H), 1.58-1.55 (PLLA, d, CH_3, 3H), 1.41 (PS, s, backbone CH_2, 2H). These results align with the chemical structures of PS-b-PLLA and PS-b-PDLA shown in Figure S5. The relative volume fractions of PS and polylactide blocks of the BCPs* were determined by comparing the relative intensities of the characteristic peaks corresponding to the PS and PLLA or PDLA blocks.

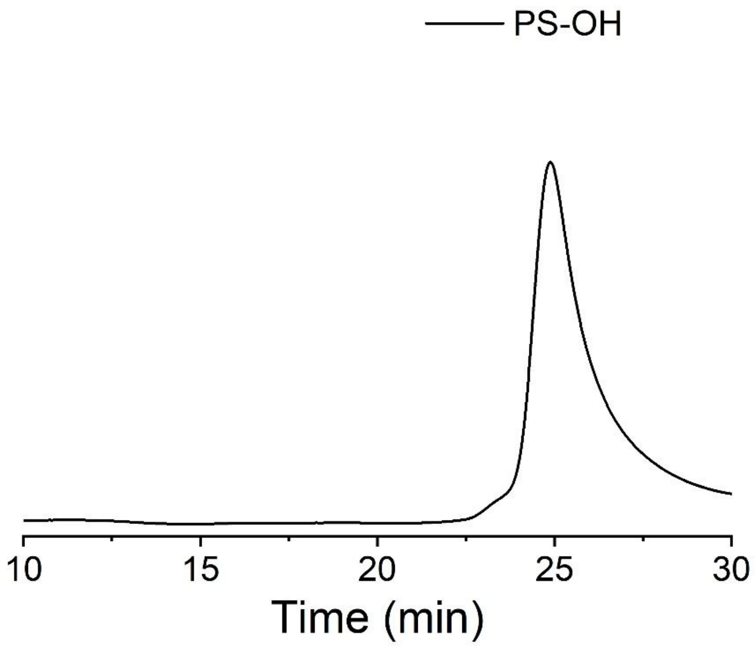

Figure S3. The GPC traces of macroinitiator PS-OH.

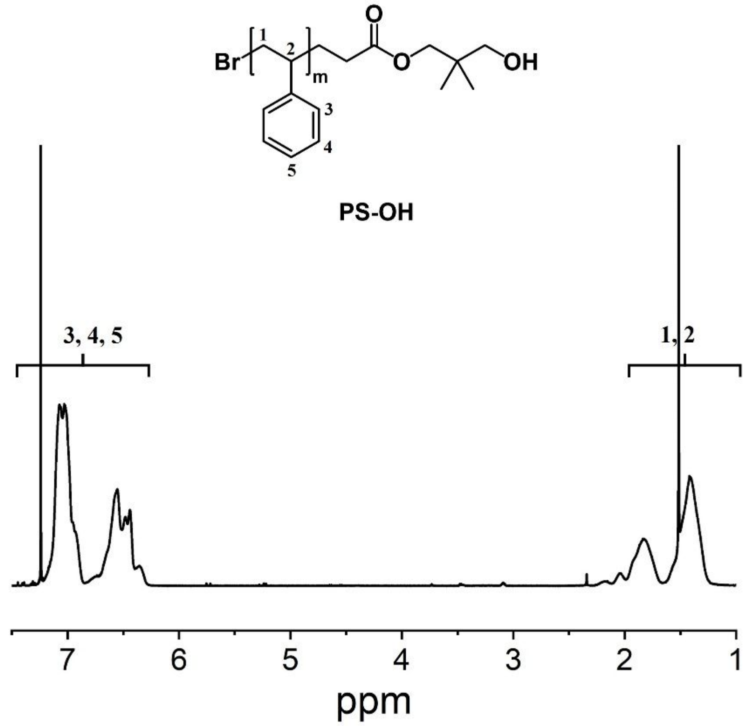

Figure S4. ^{1}H NMR spectrum (500 MHz) of PS-OH.

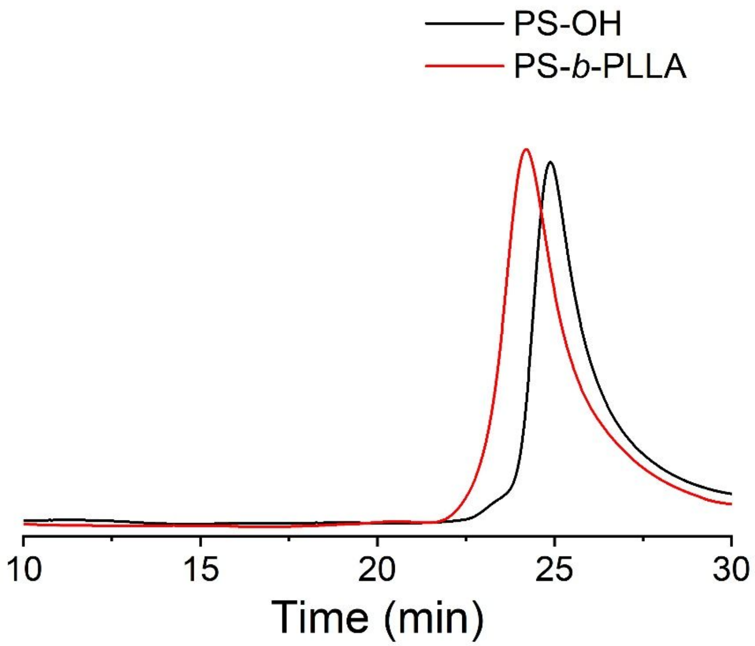

Figure S5. Comparison of GPC curves between PS-OH, PS-*b*-PLLA.

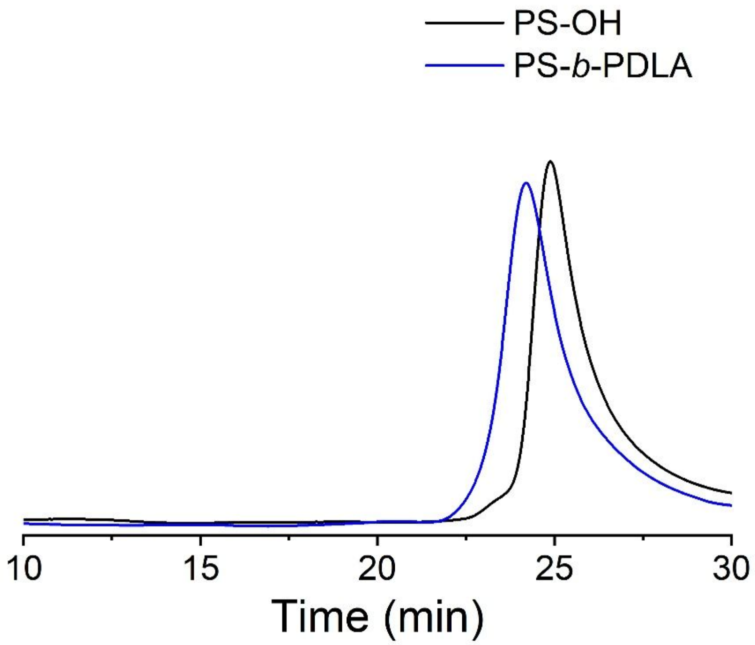

Figure S6. Comparison of GPC curves between PS-OH, PS-*b*-PDLA.

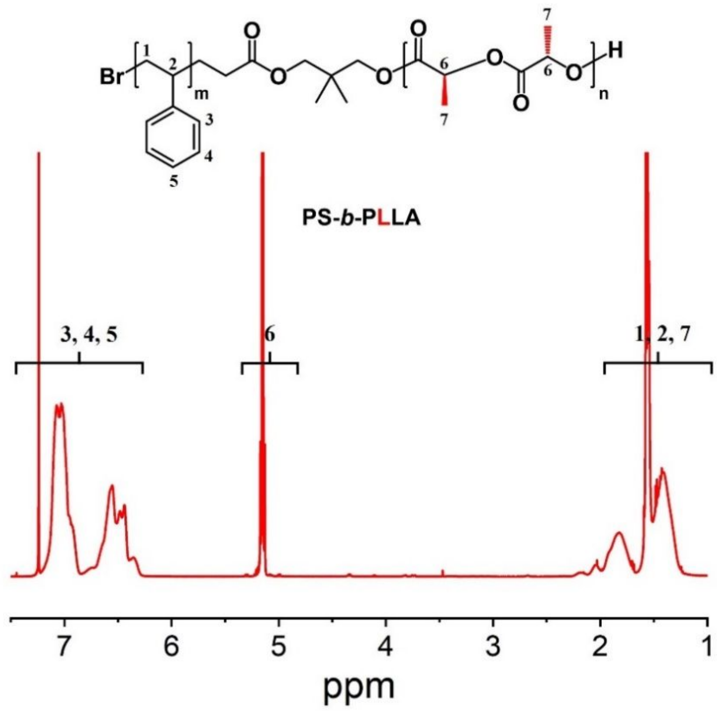

Figure S7. ^{1}H NMR spectrum (500 MHz) of PS-*b*-PLLA.

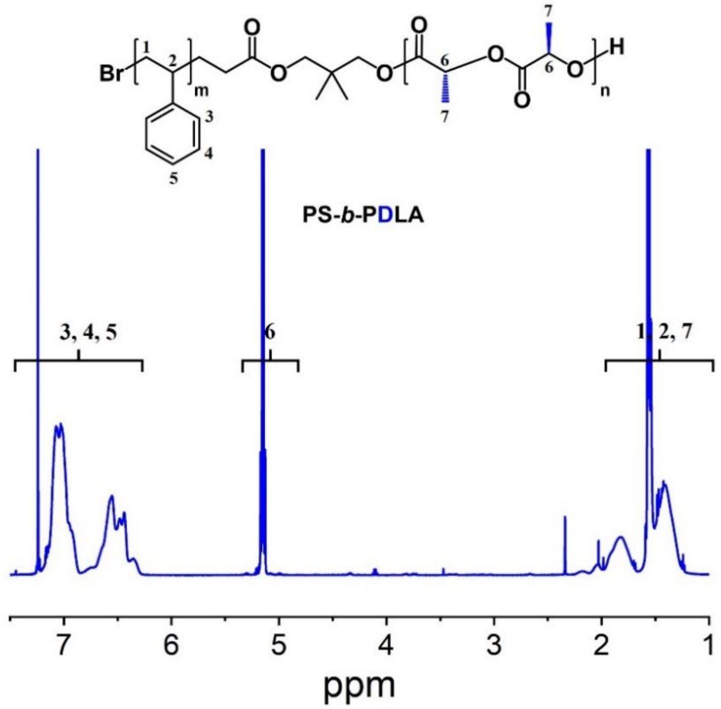

Figure S8. ^{1}H NMR spectrum (500 MHz) of PS-*b*-PDLA.

Thermal properties. The thermal properties of polylactide-based diblock copolymers were examined by thermogravimetry (TGA) and differential scanning calorimetry (DSC). TGA assesses the thermal stability and the degradation of the synthetic samples, while DSC investigates the thermal characteristics such as glass transition temperature (Tg), crystallization temperature (Tc), and melting temperature (Tm). **Figure S9** shows two degradations stages in TGA thermograms of PS-*b*-PLLA and PS-*b*-PDLA. The first degradation stage, ranging from 172 °C to 300 °C, is attributed to the PLLA or PDLA block. The second degradation stage, ranging from 350 °C to 450 °C, is attributed to the PS block. Specifically, the weight loss for the first and second degradation stages is approximately 30% and 70%, respectively, in line with the results obtained from ^{1}H NMR spectra.

Figure S10 show that there are two T_g in the DSC thermograms of PS-*b*-PLLA and PS-*b*-PDLA. The first T_g are observed at 51.49 °C and 51.34 °C, attributed to the PLLA and PDLA segments, respectively, while the second T_g are observed at 92.30 °C and 92.29 °C, attributed to the PS segments in PS-*b*-PLLA and PS-*b*-PDLA, respectively. Note that no exothermic peaks from the crystallization or endothermic peaks from the melting can be found in **Figure S10**, suggesting that the crystallization effect would not significantly affect the mesochiral self-assembly *via* microphase separation.

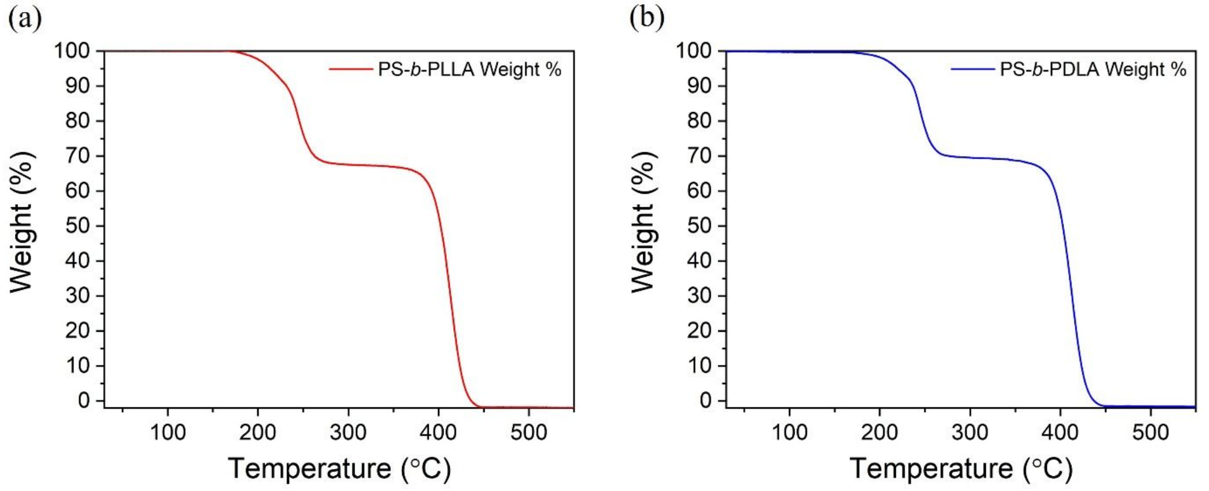

Figure S9. TGA thermograms of (a) PS-*b*-PLLA, (b) PS-*b*-PDLA. The heating rate is 10°C/min.

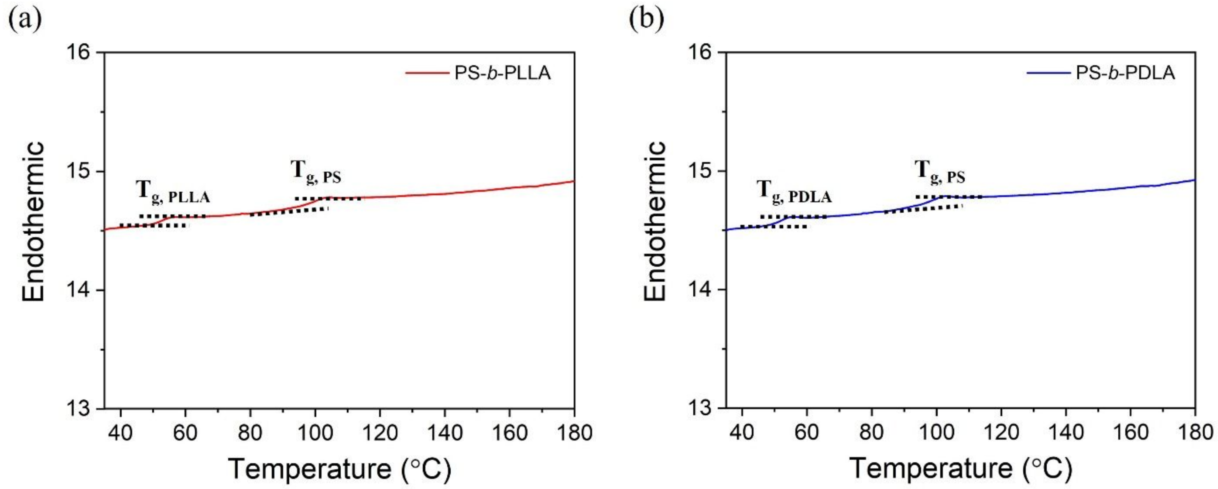

Figure S10. DSC thermograms of (a) PS-*b*-PLLA, (b) PS-*b*-PDLA. The heating rate is 10° C/min.

Instrumentation

Electronic Circular Dichroism Spectroscopy (ECD). The ECD spectra were measured on JASCO J-815 spectrometer. The CD spectra and the UV-Vis absorption spectra could be obtained simultaneously during measurements at scanning rate of 100 nm/min with 5~10 accumulations. Solution-state samples were prepared in a cylindrical quartz cuvette with light path-length of 1.0 mm whereas solid-state samples were casted on the quartz plate. The unit of CD intensity from spectrometer commonly reported as ellipticity (θ). The parameter of $\Delta\varepsilon$ for solution state was evaluated by equation 1 and the parameter of A for solid state was evaluated by equation 2, in which c is the concentration, l is path length, I_0 is the intensity of light impinging on the cell, and I is the intensity after passing through the sample.

$$\Delta\varepsilon = \frac{CD\ (mdeg)}{(32980 \times c \times l)} \quad (1)$$

$$A = log\frac{I_0}{I};A = \ \varepsilon cl \quad (2)$$

Vibrational Circular Dichroism Spectroscopy (VCD). The VCD spectra were measured on JASCO FVS-6000 spectrometer. The VCD spectra and the IR absorption spectra could be obtained simultaneously during measurements at a resolution of 4 cm^{-1} with 4500~6000 accumulations. Solution-state samples were prepared in a cylindrical BaF_2 cuvette with a path length of 50 μm whereas solid-state samples were casted on the Si wafer. The baseline was calibrated using (+)/(−)α-pinene as a standard.

Circular Polarized Luminescence Spectroscopy (CPL). The CPL spectra were measured on JASCO CPL-300 spectrometer. The CPL spectra and the photo-luminescence spectra could be obtained simultaneously during measurements. Solution-state samples were prepared in a cylindrical quartz cuvette with light path-length of 1.0 mm whereas solid-state samples were casted on the quartz plate.

The dissymmetry factor (g_{lum}) of the circular polarized luminescence at the excited state was evaluated as $g_{lum} = 2(I_L - I_R)/(I_L + I_R)$, in which I_L is the intensity of the signals for *l*-CP light, I_R is the intensity of the signals for *r*-CP light, under the incident UP light.

Transmission electron microscopy (TEM). The transmission electron microscopy (TEM) images were obtained using JEOL JEM-2100 LaB_6 transmission electron microscope with an accelerating voltage of 200 kV. For sample preparation, the bulk samples were sectioned at room temperature by Leica Ultra-microtome and collected on copper grids (100 mesh). Subsequently, the sample of PS-*b*-PLLA or PS-*b*-PDLA was stained by exposing the samples to the vapor of RuO_4 for 3 hours. The RuO_4 would interact with PS blocks, giving the bright PLLA or PDLA microdomains in dark PS matrix in TEM *via* mass contrast. For the sample of PS-*b*-PLLA/$(Eu(fod)_3)_{0.1}$ or PS-*b*-PDLA/$(Eu(fod)_3)_{0.1}$, due to the presence of heavy metal, the dark PLLA or PDLA microdomains in bright PS matrix can be clearly observed without staining.

Small-angle X-ray scattering (SAXS). Small-angle X-ray scattering (SAXS) experiments were examined at the synchrotron X-ray beam-line TLS 23A and TPS 13A at the National Synchrotron Radiation Research Center (NSRRC). SAXS data were collected with a 15-keV X-ray beam and a sample-to-detector distance of 10 m. One-dimensional (1D) linear profile was obtained by integration of the 2D pattern. The scattering intensity profiles were plotted with the scattering intensity (I) versus the scattering vector (q), where $q = 4\pi\lambda^{-1}\sin\theta$ defined by the scattering angle 2θ and X-ray wavelength λ (0.8266 Å used).

Sample preparation

ECD measurements. Solution-state samples were prepared by dissolving sample in dichloromethane (DCM) at a concentration of 0.5 wt%. Solid-state samples were prepared by spin-coating a 5 wt% of sample in 1,1,2-trichloroethane (TCE) solution on the quartz plate. To acquire well-ordered self-assembled morphologies from microphase separation, the solid-state samples were prepared by solvent vapor annealing in the saturated dichloromethane (DCM) vapor atmosphere for 3 days.

VCD measurements. Solution-state samples were prepared by dissolving sample in *d*-chloroform ($CDCl_3$) at a concentration of 4 wt%. Solid-state samples were prepared by dip-coating a 12 wt% of sample in chloroform ($CHCl_3$) solution on the Si wafer. To acquire well-ordered self-assembled morphologies from microphase separation, the solid-state samples were prepared by solvent vapor annealing in the saturated dichloromethane (DCM) vapor atmosphere for 3 days.

CPL measurements. Solution-state samples were prepared in 2 wt% sample in dichloromethane (DCM) solution. Solid-state samples were prepared by dip-coating a 12 wt% of sample in chloroform ($CHCl_3$) solution on the quartz plate. To acquire well-ordered self-assembled morphologies from microphase separation, the solid-state samples were prepared by solvent vapor annealing in the saturated dichloromethane (DCM) vapor atmosphere for 3 days.

Preparation of self-assembled bulk samples. Self-assembled bulk samples were prepared by solution casting from DCM solution. At first, the sample was dissolved in DCM at a concentration of 10 wt%, then, the solution was transferred in a vial and sealed well by aluminum foil having holes for the slow evaporation of the solvent to acquire the formation of well-ordered phases from self-assembly.
